# Appendicular Muscle Physiology and Biomechanics in *Crocodylus niloticus*

**DOI:** 10.1093/iob/obaa038

**Published:** 2020-11-05

**Authors:** Krijn B Michel, Tim G West, Monica A Daley, Vivian R Allen, John R Hutchinson

**Affiliations:** 1 Department of Comparative Biomedical Sciences, Structure and Motion Laboratory, Royal Veterinary College, North Mymms, Hawkshead Lane, Hertfordshire, AL9 7TA, UK; 2 Department of Ecology and Evolution, University of California, Irvine, CA, 94704, USA

## Abstract

Archosaurian reptiles (including living crocodiles and birds) had an explosive diversification of locomotor form and function since the Triassic approximately 250 million years ago. Their limb muscle physiology and biomechanics are pivotal to our understanding of how their diversity and evolution relate to locomotor function. Muscle contraction velocity, force, and power in extinct archosaurs such as early crocodiles, pterosaurs, or non-avian dinosaurs are not available from fossil material, but are needed for biomechanical modeling and simulation. However, an approximation or range of potential parameter values can be obtained by studying extant representatives of the archosaur lineage. Here, we study the physiological performance of three appendicular muscles in Nile crocodiles (*Crocodylus niloticus*). Nile crocodile musculature showed high power and velocity values—the flexor tibialis internus 4 muscle, a small “hamstring” hip extensor, and knee flexor actively used for terrestrial locomotion, performed particularly well. Our findings demonstrate some physiological differences between muscles, potentially relating to differences in locomotor function, and muscle fiber type composition. By considering these new data from a previously unstudied archosaurian species in light of existing data (e.g., from birds), we can now better bracket estimates of muscle parameters for extinct species and related extant species. Nonetheless, it will be important to consider the potential specialization and physiological variation among muscles, because some archosaurian muscles (such as those with terrestrial locomotor function) may well have close to double the muscle power and contraction velocity capacities of others.

## Abstract (es)

Les archosaures, le groupe de reptiles incluant les oiseaux et les crocodiles actuels, sont caractérisés par une diversification importante de leurs formes et fonctions locomotrices depuis le Trias il y a environ 250 millions d’années. Des études biomécaniques et musculaires centrées sur les membres appendiculaires sont donc essentielles pour comprendre le lien qui unit les fonctions locomotrices des archosaures avec leur histoire évolutive et leur forte diversité. Les données les plus fréquemment utilisées, telles que la vitesse de contraction et la force musculaire, ne sont pas accessibles pour les archosaures éteints tels que ceux issus de la lignée fossile des crocodiles (pseudosuchiens), les ptérosaures ainsi que les dinosaures non-aviens. Ces données sont pourtant nécessaires à l’établissement de modélisations et de simulations biomécaniques à l’échelle du groupe. Il est cependant possible d’obtenir une estimation de ces paramètres à partir des archosaures actuels. Cette étude présente en détails la physiologie de trois Crocodiles du Nil (Crocodylus niloticus) en détaillant les performances musculaires de leur appareil locomoteur. Les muscles des Crocodiles du Nil présentent des forces et des vitesses de contraction élevées. Les performances du muscle flexeur tibialis internus 4, qui est un petit muscle ischio-jambier entre la hanche et le genou fréquemment sollicité chez les animaux terrestres, s’avèrent être particulièrement élevées. Notre étude met en évidence des différences de physiologie entre les muscles, potentiellement liées aux différences de fonctions locomotrices et à la composition des différents types de fibres musculaires. En couplant ces nouvelles données avec celles déjà connues chez les oiseaux, il est possible de mieux estimer les paramètres musculaires d’espèces éteintes ainsi que d’espèces actuelles phylogénétiquement proches. Il est également essentiel de considérer les différentes spécialisations ainsi que les variations de physiologie musculaire. En effet, les muscles de certains archosaures, en particulier ceux dotés d’un mode de locomotion terrestre, pourrait présenter des forces et vitesses de contractions musculaires bien supérieures à celles d’autres espèces.

By Romain Pintore, RVC

## Introduction

The clade Archosauria originated very close to the Permian-Triassic boundary approximately 250 million years ago, and rapidly diversified during the Triassic period. Numerous morphologies and presumed locomotor modes evolved during the Triassic diversification of Archosauria, and further divergence evolved after the Triassic–Jurassic mass extinction event (Charig 1972; Sereno 1991; Hutchinson and Gatesy 2000; Padian et al. 2010). The quadrupedal Crocodylia lineage (Pseudosuchia) ultimately became restricted to a more amphibious lifestyle and retained (or even re-acquired) some plesiomorphic locomotor features for Archosauria (Parrish 1987). In contrast, the origin of birds (Aves) from bipedal, nonavian dinosaurs occurred at or close to the origin of flight, enabling an adaptive radiation (Chiappe and Dyke 2002). Although much of this archosaurian locomotor disparity is evidenced by skeletal morphology, it has long been recognized that major changes in muscle form and function accompanied skeletal changes (Charig 1972; Hutchinson and Gatesy 2000; Hutchinson 2001).

Muscle form and function are linked not only by anatomy and motor control but also by contractile physiology and biomechanics at the level of sarcomeres and muscle fibers. Detailed analysis of the physiological attributes of muscles as parts of the musculoskeletal lever system may reveal characteristics that contribute to the locomotor performance of that system. Yet evolutionary changes (or conservatism) in the intrinsic properties of locomotor musculature have barely been considered in the context of the evolution of Archosauria. The force per unit area (maximal isometric stress; σ), contractile velocity, power, force–length (F–L) relationship, and other metrics are critical determinants of muscle function (Zajac 1989; Medler 2002; Millard et al. 2013) and thus a synthesis of new and existing data for archosaurian appendicular muscles is timely. The aim of this study is therefore to characterize performance in muscles involved in terrestrial locomotion within living representatives of the pseudosuchian archosaur lineage.

Extant crocodylians are the only living tetrapods to use nearly the full range of recognized quadrupedal terrestrial locomotion patterns, from highly abducted, laterally undulating “sprawling” gaits to more erect high-walking or even asymmetrical galloping and bounding gaits in some species (e.g., Webb and Gans 1982; Gatesy 1991). Nile crocodiles (*Crocodylus niloticus*) are known to use this full repertoire (Cott 1961), unlike alligators (e.g., Allen et al. 2014; Hutchinson et al. 2019), and breed well in captivity so they are accessible for experimental studies. The only studies of crocodylian muscle physiology and biomechanics directly relevant to this study involved the main locomotor muscle connecting the tail to thigh (M. caudofemoralis longus; Seebacher and James 2008) and the ontogeny of the jaw adductor system (Gignac and Erickson 2016). Thus crocodylian locomotor muscle physiology is not well-studied despite their wide variety of locomotor modes.

In contrast, the terrestrial locomotor repertoire in extant birds is relatively conservative, characterized by an erect, adducted hindlimb posture, crouched hindlimbs, and a largely parasagittal gait (e.g., Gatesy and Biewener 1991; Daley and Birn-Jeffery 2018). Many birds have largely retained ancestral avian traits such as a cursorial limb morphology and a preference for running vs. flight when disturbed. Species with this more terrestrial locomotor mode and associated cursorial specializations include typical galliforms (chickens, turkeys, and kin; widely studied for muscle physiology and biomechanics) and Palaeognathae (ostriches, emus, tinamous, and kin).

Prior studies of appendicular muscle physiology in galliforms and other members of the Neognathae radiation inform our estimates of ancestral muscle performance (e.g., Reiser et al. 1996, 2013; Askew and Marsh 2001; Medler 2002; Seebacher and James 2008; Cuff et al. 2019). Hence avian locomotor muscle physiology is relatively well known compared with crocodylian muscles. These existing studies of avian muscle physiology enable us to begin to bound the range of performance potential in locomotor muscles for models and computer simulations of extinct ornithodiran archosaurs (i.e., birds and their closer relatives), but a robust phylogenetic bracket for all Archosauria requires more physiological data from Crocodylia.

The appendicular osteology and myology of Nile crocodiles and other Crocodylia have been fairly well-described (e.g., Cong et al. 1998; Meers 2003; Allen et al. 2014), and some muscle activation patterns during gait are also known (Gatesy 1997; Cuff et al. 2019). However, to properly compare locomotor dynamics between animals, information on physiological limits of the motors that power these biomechanical systems is essential. Such data are important not only for basic understanding of the evolutionary adaptations of individual muscles, but also as inputs for computational analyses of locomotor biomechanics (Zajac 1989; Bates and Schachner 2012; Millard et al. 2013; Rankin et al. 2016). While vertebrate skeletal muscles tend to have relatively conserved physiological properties compared to many other metazoans (Medler 2002), further sampling throughout poorly studied taxa such as Crocodylia will better inform the inherent assumptions required for musculoskeletal modeling and simulations. Full characterization of all archosaurian muscles is not feasible, but qualitative sampling will allow us to explore possible specialization or conservatism of certain muscle properties, ideally relative to their function. To that end, we acquired fresh Nile crocodile muscles from captive-raised animals and tested the performance of a variety of different muscles *ex vivo*. We also obtained immunohistochemical data from these muscles to qualitatively assess their fiber type composition. These data allowed us to investigate the performance range of whole muscle fiber bundles and to what extent this range is comparable between the two lineages of extant archosaurs. We discuss how these data on muscle physiology and mechanical performance compare with other such published data for Aves and (to the limited extent that data exist) for Crocodylia.

## Materials and methods

### Animals

Captive-bred and raised Nile crocodiles (*C. niloticus*) were obtained from registered breeders and suppliers (La Ferme aux Crocodiles, Pierrelatte, France). The animals were housed on the Hawkshead campus of the Royal Veterinary College, with approval from the UK Home Office and Royal Veterinary College Ethics Committee, for usage in this and other studies. The animals were euthanized according to supplementary material Appendix 1 of Schedule 1 (revised 1997) of the Animals (Scientific Procedures) Act 1986. Nine Nile crocodiles (all female, juveniles ∼1–2 years old) were used for the experiments, body masses ranged from 1.38 to 6.96 kg.

**Fig. 1:**
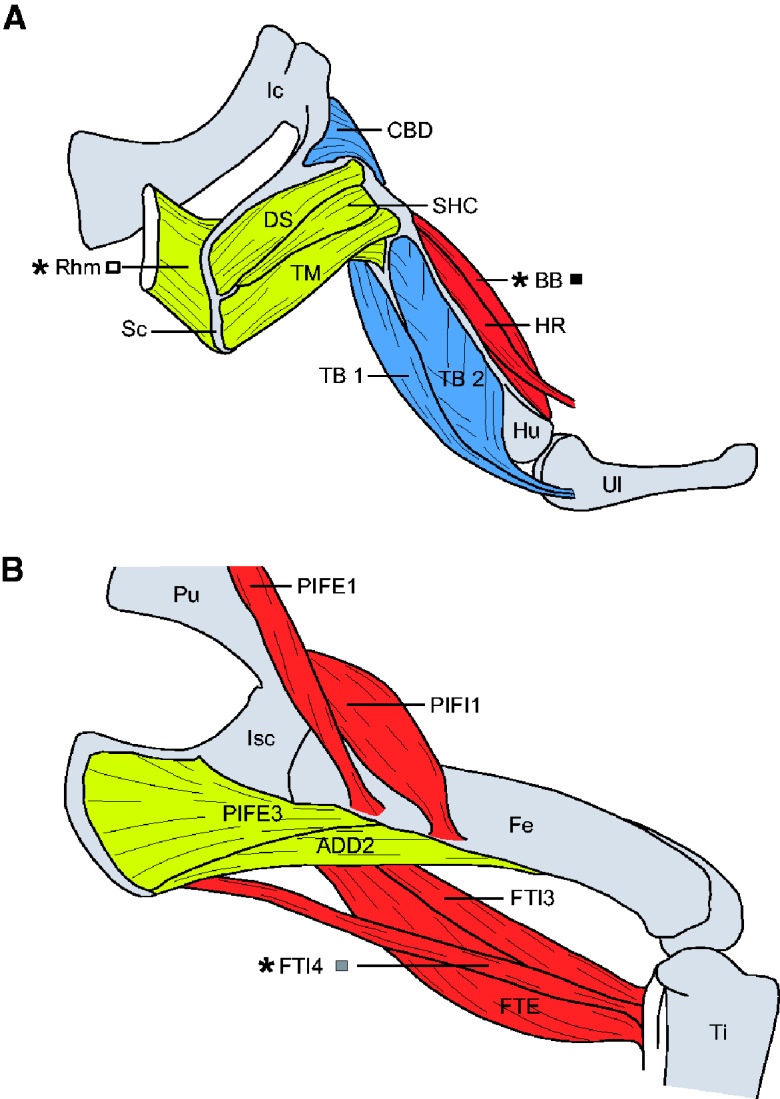
Schematic overview of the anatomical position of several appendicular muscles in the Nile crocodile. Forelimb in A: CBD, Coracobrachialis brevis dorsalis; DS, Deltoideus scapularis; HR, Humeroradialis; Hu, Humerus; Ic, Interclavicle; SHC, Scapulohumeralis caudalis; TB 1, Triceps brevis intermedius; TB 2, Triceps brevis cranialis; TM, Teres major; Ul, Ulna. Hindlimb in B: ADD2, Adductor 2; Fe, Femur; Isc, Ischium; PIFE1, Pubo-ischio-femoralis externus 1; PIFE3, Pubo-ischio-femoralis externus 3; PIFI1, Pubo-ischio-femoralis internus 1; Pu, Pubis; Ti, Tibia. ^∗^Muscle names (Rhm, BB, and FTI4) denote muscles studied here; grayscale squares indicate coding used for data points in other figures.

Muscles were selected to allow comparison between forelimb and hindlimb musculature, as well as limb and body/scapula (Sc) support musculature. Each of the appendicular muscles was chosen in part for practical reasons, based on quick accessibility via dissection, and on their suitability for mounting in the *ex vivo* muscle lever system (e.g., small size and good tendinous/cartilaginous attachments to the ends of the lever mechanism). Unfortunately, muscles with strict homology within the archosaurs lineages could not be chosen due to mounting restrictions. We, therefore, focused on muscles that were analogous in function to more generally known limb extensors and flexors, and used during terrestrial locomotion. This led us to selected the following muscles: a presumed scapular elevator M. rhomboideus (Rhm), a forelimb protractor M. biceps brachii (BB), and a hindlimb hip extensor and knee flexor M. flexor tibialis internus 4 (FTI4) (Fig. 1 and Table 1).

**Table 1:** Summary of the origins and insertions of the muscles used in our muscle physiology experiments

Species	Muscle	Origin	Insertion	Action
Nile crocodile	Rhm	Fascia covering the eighth and ninth dorsal vertebrae	Dorsal portion of the medial Sc-coracoid	Pectoral girdle adductor, elevator, and stabilizer
	BB	Cranial edge of the coracoid shaft	Caudal aspect of the proximal radius	Shoulder flexor and elbow flexor
	FTI4	Fascia near latero-ventral ilium	Proximo-(caudo)medial tibia via tendon	Hip extensor and knee flexor

Anatomical position and presumed action based on literature (Cong et al. 1998; Meers, 2003; Allen et al. 2014).

### Muscle fiber bundles

Bundles of intact muscle fibers were dissected from the freshly euthanized crocodiles. We took samples from the edge of the muscle to include tendon or aponeurosis at ends for anchoring to the experimental apparatus, and cut away extraneous connective tissue or fat. These bundles were cut so that fibers aligned longitudinally with the force transducer; hence our measurements avoided complications from muscle pennation, a higher-level phenomenon in muscle models (e.g., Zajac 1989). Pennation is absent anyway in the FTI4 and Rhm, and variable ∼0–30° in the BB (Allen et al. 2014). The muscle bundles were removed within 30 min of euthanasia and kept in physiological saline (mmol L^−1^: NaCl 135, KCl 4.0, CaCl_2_ 2.35, MgCl_2_ 0.85, NaH_2_PO_4_ 1, and NaHCO_3_ 20) and glucose 5.5 mmol L^−1^ and equilibrated with 95% O_2_, 5% CO_2_ (Curtin et al. 2015). During dissection, the bundles were regularly moistened with physiological saline. Preparation of muscle bundles for the mechanics measurements involved dissection of muscle strips from the preparation until the remaining bundle was small enough (in cross-section) to not saturate the 500 mN signal capacity of the force transducer used (Series 300B Lever Arm System, Cambridge Technology Inc., Watertown, MA). Our reduced muscle preparations were necessary because larger muscle preparations would have been oxygen-limited, introducing potentially severe artifacts into measurements (Barclay 2005; Allen et al. 2008). The average cross-sectional area (CSA) of the muscle bundles was 1.7 mm^2^ ± 0.56 standard deviation (SD) (averages FTI4 1.3, Rhm 1.6, and BB 2.1; range 0.7–2.9 mm^2^). Some of the dissected strips were immediately immersed into ice-cold 2% Triton-X 100 (30 min), made up in a relaxing solution (containing in mM, except where stated; imidazole 6, magnesium acetate 8, potassium propionate 70, Na_2_-ATP 7, ethylene glycol-bis(2aminoethylether)-*N*, *N*, *N'*,*N'*-tetraacetic acid 5, and leupeptin mg L^−1^, trypsin inhibitor 50 mg L^−1^). After being washed in Triton-free relaxing solution (30 min, on ice), these “skinned” muscle strips were stored at −20°C in relaxing solution made up in 50% glycerol. These preparations served a dual purpose; first, as source material to approximate the slow and fast myosin heavy chain (MHC) content (see below) of the larger bundles used for mechanics tests and, second, for later dissection and mechanics testing of single fibers.

Experiments on the intact muscle bundles were done at 25°C. The crocodiles were housed at around this temperature (details in Cuff et al. 2019); as ectotherms in the wild, they would encounter comparable or higher temperatures varying geographically and temporally, but for the purposes of our experiment, a single controlled temperature was necessary. The ends of the muscle bundles were connected to the setup by a clip or suture at the tendon or cartilage terminus. One end of the muscle bundle was attached to a fixed hook and the other to the motor/force transducer (Series 300B Lever System, Cambridge Technology Inc., Watertown, MA, USA). Electrical pulse stimulation of the bundle was generated using a purpose-built device. The force, length, and stimulus pattern were controlled using Aurora Scientific (Aurora ON, Canada) DMC software version 5.321 interfaced with a National Instruments (Austin, TX, USA) USB-6229 DAQ device. Data were acquired at 10 kHz.

The maximum isometric muscle force and the accompanying optimal muscle length were determined by a series of short contractions (1 s duration each) at pre-set muscle lengths. The active force (mN) production of each muscle was measured over a range of muscle lengths to encompass contraction at a more stretched or slack state. This allowed us to determine broad F–L relationships for each bundle and the fiber length (*L*_0_) at which maximum isometric force (*F*_IM_) was produced. The 1 s isometric contractions were evoked using a stimulus pattern that generated bi-directional pules (1 ms duration) at 200 Hz. Active force was calculated as the difference between peak total force and passive force (i.e., without muscle stimulation).

Measurements of muscle force and length-change during shortening contractions were made with the 300B system in “force control” mode (e.g., Curtin et al. 2015). Briefly, the Aurora DMC software was set up to stimulate the muscle to achieve peak isometric force and then, at a pre-set point in the time course (e.g., 500 ms after the stimulus onset in Fig. 3), a predetermined sub-maximal level of force was achieved, and then held, by a rapid step-change in length, followed immediately by a stable rate of fiber shortening. The muscle length-change needed to clamp the force at the new level was recorded (Fig. 3). In these experiments, stimulation was stopped while the system was still in force–control mode, so the muscle relaxed from the clamped level of force. We waited 2 min between stimulations. The force and length-change time-courses for a range of force-clamps were used to calculate relative forces (force during shortening divided by maximum isometric force) and shortening speed (muscle-lengths per second, units of s^−1^).

It was our aim to focus on defining the margins of the F–L plateau regions rather than to generate and scrutinize complete F–L relationships. We purposely changed passive muscle lengths within a limited range, achieving active forces within 50% of peak isometric force. The optimal muscle length (L_0_) for assessing muscle power–force and force–velocity (F–V) relationships were chosen from the plateau region of F–L.

For stimulations made with force-clamps, each force control event was pre-set to initiate at a specific time after the onset of the stimulus, after the preparation had achieved stable isometric force. Measurements of shortening velocity were limited to overall length-changes (i.e., including the step-down of length occurring during an initial step-down of force) between 1.0 L_0_ and 0.9 L_0_, even at the fastest shortening speeds that resulted from the lowest levels of force-clamp. This meant that measurements of the rate of length-change and the stable level of clamped force were made for short intervals (e.g., ranging 10–30 ms), initiated as soon as possible after the step-decrease of force, and length, from the isometric state.

Normalized power values were calculated as the product of relative force and shortening speed. Power–Force relationships were generated for each series of force clamps and these were fit (details below) in order to evaluate *Q*_max_ (peak normalized power, in units s^−1^). Normalized power is appropriate for fitting purposes as it is independent of changes in muscle isometric force that may occur through a series of activation–relaxation cycles.

A test of the rate of muscle bundle fatigue was done after the measurements for F–L and F–V relationships were completed. This was achieved by subjecting the muscle bundle to a series of brief (1 s duration) maximum isometric stimulations in rapid succession (1 s between stimulus trains). The rate of fatigue was taken as the number of contractions for force to drop to 50% of *F*_IM_ recorded at the start of the series.

At the conclusion of the experiments, the fiber bundles were removed from the setup and pinned onto strips of Sylgard 184 (Fisher Scientific, UK) at *L*_0_ and fixed with 70% alcohol in order to dissect whole fibers and measure resting fiber length. Extraneous materials, such as tendons and cartilage, were then carefully removed from the fixed muscle fiber preparation, after which the preparation was re-hydrated in water, blotted, and weighed. Volume of the muscle fiber preparation was calculated from the blotted mass, assuming a density of 1.064 at 25°C (Mendez et al. 1960), and CSA estimated as the ratio of bundle volume: fiber length.

### Data normalization

**Fig. 2:**
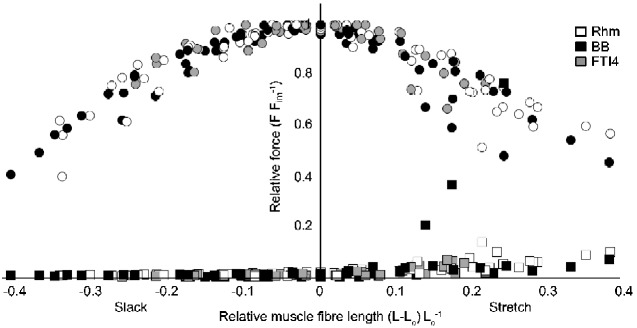
The force (F) – length (L) relationship for all measured Nile crocodile muscles. The muscle fiber length L on the *X*-axis was normalized by subtracting L_0_ (i.e., 0 = L_0_). The force F on the *Y*-axis was normalized by F_IM_. Each set of grayscale circles shows the force produced by a muscle across a range of lengths. Each matching grayscale square shows the passive muscle force. The normalized “Hill-type” F–L relationship can be seen across the different muscles (see Legend for grayscale coding).

To allow comparison between muscles and between animals some values were normalized as follows: Force (mN) values were divided by the corresponding *F*_IM_ of that muscle (as in the *Y*-axis in Fig. 2). Velocity data (mm s^−1^) were normalized by L_0_; the units were therefore s^−1^. Power for power–force curve-fitting and deriving values of peak power was also presented as normalized power, as described below.

### Curve-fitting for maximum power

Normalized power (Q, s^−1^) and normalized force (F, dimensionless) were determined for each muscle bundle from the data obtained from the force and velocity values measured in a series of different force-clamps. Normalized force (F) was simply the ratio of clamped force to the isometric force measured in the same stimulation. Normalized power (Q) was the product of Force and velocity in units s^−1^ for each different force-clamp. Generally, 10–13 different force-clamps were necessary in order to populate a plot of Q and F with sufficient data, across a range of F values between 0 and 1 (e.g., see results Fig. 4). The methods for power–force curve-fitting were based on Curtin et al. (2015). Their key equation for fitting power–force curves to values of Q vs. F is:

(1) $$Q=(Q_{\max}F_{O}^{*}/F_{Q_{\max}}^{2})\cdot (F(1–(F/F_{O}^{*}))/(1+F((F_{O}^{*}–2F_{Q_{\max}})/F_{Q_{\max}}^{2}))$$

To find the best fit to Hill’s equation, three parameters were adjusted for the series of Q and F values measured for each muscle sample tested: the maximum normalized power (*Q*_max_), the relative force at the maximum normalized power (*F_Q_*_max_), and the force intercept on the power vs. force curve (F*_o_). Key muscle properties that can be derived from the fitted values include the velocity at maximum power (*V_Q_*_max_ = *Q*_max_/*F_Q_*_max_) and the maximum shortening velocity of the preparation (*V*_max_ =  (*Q*_max_ · F*_o_)/*F*_max_^2^).

The maximum power per unit volume (L) in W L^−1^ is the product of *Q*_max_ and the maximum isometric stress generated by the muscle preparation:

(2) $$Q_{\max}F_{\mathrm{IM}}{\;L}_{o}/\mathrm{CSA}{\;L}_{o}=Q_{\max}F_{\mathrm{IM}}/\mathrm{CSA}$$

Where CSA is the cross-sectional area of the preparation in mm^2^.

### Statistics

We used linear mixed-effects (LMEs) models and Analysis of Variance (ANOVA) tests to determine how much of the variation in our data was attributable to muscle ID (Rhm, BB, and FTI4) or accountable to specific covarying factors. The several LME models tested for differences in each measure of maximum power, peak stress (isometric σ and stress at peak power), and velocity (*V*_max_ and velocity at peak power) among muscle IDs while accounting for potential effects of variation caused by muscle fatigability, muscle length and body mass (Supplementary data, Table S1). Muscle ID was the predictor variable, or the primary factor of interest, and a categorical fixed factor. Fatigability (number of stimulations until 50% original isometric stress remains) and total muscle length were continuous, secondary covariates with potential to confound the effect of muscle ID, so we included these as continuous fixed effects. The variation attributable to body mass was also quantified by including it as a continuous fixed factor in the model. Each LME model was evaluated using the Akaike Information Criteria (AIC) and coefficient of determination (*R*^2^) to compare models with and without body mass and selected the model with the lowest AIC value and highest *R*^2^ (as per Nakagawa and Schielzeth 2013); in all cases, these two criteria concurred. Similar to Gordon et al. (2015), we used the AIC as a method of comparing the goodness of fit of the models, while penalizing those with more parameters and promoting parsimonious model selection. The final LME model reported here was, therefore, ensured to characterize the variance in the data with the simplest model possible. We used ANOVA *F*-statistics to test the influence of each factor (muscle ID, fatigability, muscle length, and body mass) on each individual output measure (e.g., power, stress, and velocity). The threshold for a significant difference between muscles and factors was set at *P* ≤ 0.05 by the 95% confidence intervals for differences between categories.

### Fiber typing using immunohistochemical staining

Small fragments (up to 1 cm long, 2–3 mm thick) of the skinned muscle strips were taken for assays of Types I and II MHC-I and MHC-II, using fluorescent antibody binding to thin cryostat sections. Each muscle fragment was washed in fresh relaxing solution (30 min on ice) to remove glycerol contained in the −20°C storage solution. These were placed in separate cryomolds (Sakura, NL) that were filled with Tissue-Tek OCT embedding compound (Sakura, NL). The muscle fragments were frozen by immersion of the filled cryomolds into liquid nitrogen.

Muscle bundle sections (8–10 μm thick) were cut while frozen using a cryostat (Bright Instruments, UK) and mounted on glass superfrost plus slides (Fisher Scientific, UK). These bundles were cut from the larger bundles used for mechanical testing. Hence the fragments were likely representative of the fibers left on the main bundles. Consecutive sections were collected on separate slides and allowed to dry at room temperature for 30 min before storage at −80°C.

For immunostaining, the slides were first allowed to reach room temperature and then the sections were circled with an H4000 hydrophobic PAP pen (Vector Labs, UK). The sections were fixed in 1:1 methanol:acetone at −20°C for 15 min, then washed in phosphate-buffered saline (PBS), pH 7.4 (Fisher Scientific, UK)—three PBS changes were made ∼10-min period. Goat anti-collagen V (Bio-Rad, Hercules CA, USA) 1:20 was incubated with the sections for 1 h at room temperature before washing in three changes of PBS ∼10 min. This was followed by exposure to a 1:1000 dilution of donkey anti-goat AlexaFluor 488 (Invitrogen, ThermoFisher, UK) for 1 h at room temperature.

After a final wash in PBS, the sections were incubated with one of two anti-MHC antibodies. For slow MHC-I, a section was exposed to a 1:50 dilution of anti-myosin antibody, slow NOQ7.5.4D (Millipore, MA). For fast MHC-II, a consecutive section was exposed to a 1:1000 dilution of anti-fast MHC MY-32 (Abcam, UK). Sections were incubated with the two anti-MHC stains for one hour at room temperature, then washed with three changes of PBS ∼10 min. Lastly, the sections were incubated in a 1:1000 dilution of Alexa Fluor 594, donkey anti-mouse IgG secondary antibody (Fisher Scientific, UK) for 1 h at room temperature, then washed with three changes of PBS ∼10 min.

The sections were then coverslipped with Vectashield DAPI (Vector Labs, CA) to stain the nuclei before being examined with a Leica DM4000 fluorescence microscope fitted with TX2 594 nm, L5 488 nm, and A 350 nm filter sets. Images of the same area on the consecutive sections were captured using a ×10 or ×20 objective and the L5 filter for the collagen image and the TX2 filter for the muscle fiber type image. The same areas on consecutive sections were located by a landmark such as a blood vessel or a clear collagen structure.

## Results

Across our Nile crocodiles (*N* = 9), experiments were successfully executed on the following muscle bundles (and *N* = numbers of samples): Rhm (*N* = 6), BB (*N* = 5), and FTI4 (*N* = 4). In one case, two preparations of Rhm muscle were obtained from the same muscle of the same animal.

### F-L and force-hold examples

The crocodile muscles displayed classic “Hill-type” active F–L relationships (Fig. 2). The resulting partial F–L relationships were a typical shape, with broad plateau regions. Average (±SEM) L_0_ across all of the preparations was 34.9 ± 3.35 mm and a near-plateau region of maximal force encompassed approximately *L*_0_ ± 10%. Passive force increased gradually in most preparations when resting lengths exceeded ∼1.1 L_0_. One BB muscle displayed a sharp rise in passive force at lengths >1.2 L_0_. The margins of the active F–L plateau for this one preparation were, however, in line with the general ±10% shown as an approximation, and it was the shortening length-changes in this region that were most relevant to the subsequent determinations of muscle force and speed during shortening and, in turn, to the calculations of muscle power.

**Fig. 3:**
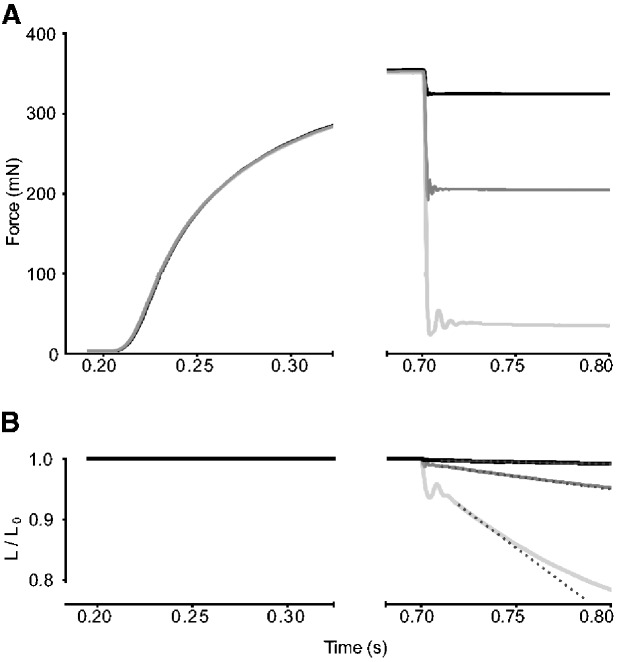
Example force-hold contractions of a BB muscle bundle in Nile crocodile. (**A**) After 200 ms, the stimulus was initiated, and the maximum isometric force was reached after 650 ms. In this example, three separate force time-courses are overlaid; force-rise was the same in each record. In the middle (dark grey) record, the targeted submaximal force was pre-set at 200 mN. A step-change of muscle length allowed the system to rapidly achieve the 200 mN target, and thereafter the stable length-change needed to hold the force stable was recorded. (**B**) Stable rates of muscle shortening (dotted lines) were determined with force clamped at different levels. Length-change was stable for 10 to 30 ms; the shortest linear periods were associated with the fastest shortening speeds attained (light grey). For clarity, the time-courses after the stimulus was stopped (at 800 ms) are not shown because the system was still holding force stable for a short period while the muscle relaxed.

**Fig. 4:**
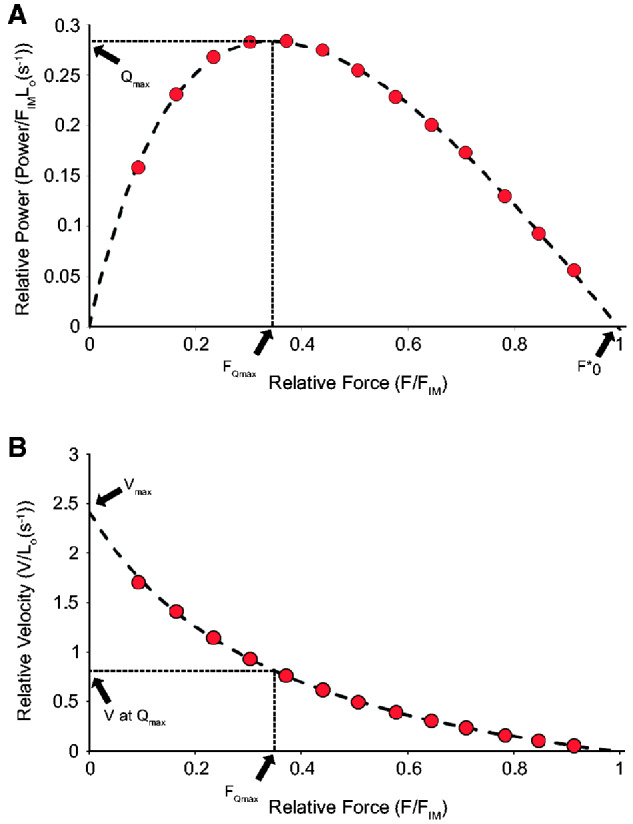
Experimentally acquired force and velocity and calculated power data from a representative Nile crocodile BB muscle bundle (same sample as in Fig. 3). (**A**) Power normalized by isometric force and L_0_ as function of force during shortening normalized by isometric force. The normalized maximum power (*Q*_max_) and the normalized force at maximum power (*F_Q_*_max_) were measured from fitting these data, normalized by *F*_IM_. (**B**) Velocity of shortening normalized by L_0_, as a function of force during shortening normalized by isometric force.

In the examples in Fig. 3, force-control began in each case at 500 ms into the stimulus time-course. The three time courses depicted overlay each other during the periods of force rise and isometric plateau, indicating that the muscles responded consistently to the maximal stimulation. For a full series with any one muscle bundle, 10–13 stimulations were required. Across the 15 muscle bundles tested, only in one case, force at the end of the simulation series was <90% of peak value measured at the beginning of the series; on average, isometric force during the final measurement in a series was 97.6 ± 0.04% of the first measurement. The low, medium, and high levels of force-clamp depicted in the example in Fig. 3 are 3 of the total of 13 different levels of force-clamp measured for this bundle of BB. The full series of force clamps were used to generate the power–force relationship shown in Fig. 4 (see also Curtin et al. 2015). As a reminder, the chief aim of fitting a power–force relationship was to extract the peak value of normalized power (*Q*_max_). Figure 4 shows values for *Q* (Equation 1), calculated for each of the 13 force clamps, plotted against values of relative force (i.e., mN during force-clamp normalized to mN of isometric force). The least-squares curve fit for *Q* as a function of relative force, as detailed in the Materials and Methods section and in Curtin et al. (2015), found values for peak normalized power (*Q*_max_), and also values for the relative (optimal) force at peak power (*F_Q_*_max_) and the x-intercept of the curve-fit, called$\;F_{O}^{*}.$ For the example of BB in Fig. 4, the normalized maximum power (*Q*_max_) was 0.284 s^−1^and the relative force at peak power (*F_Q_*_max_) was 0.342. The product of *Q*_max_ and maximum isometric stress (σ; for this preparation, 218.8 kPa) gave power in units W L^−1^ (62.1). The values shown in the example F–V plot (Fig. 4B) were derived from the outcomes of the power–force curve fitting, as described in detail in Curtin et al. (2015). The velocity at maximum power for this muscle fiber bundle was 0.83 s^−1^ and the maximum shortening velocity was 2.42 s^−1^.

**Fig. 5:**
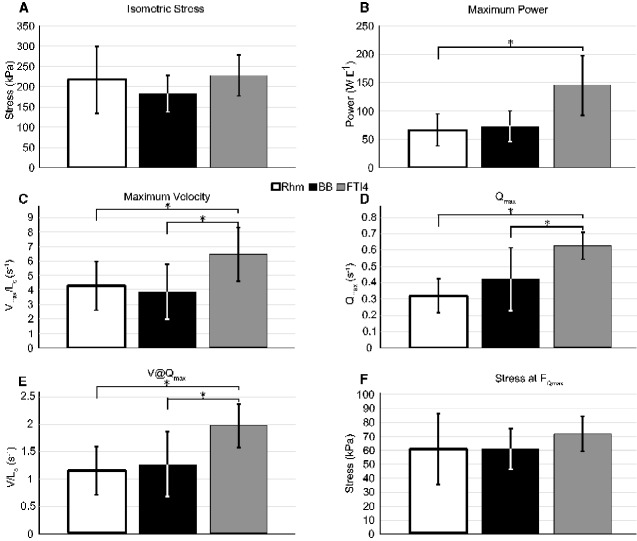
The maximum isometric stress σ (**A**), maximum power (**B**), maximum velocity (**C**) normalized power (**D**), normalized velocity at maximum power (**E**) and stress at maximum power (*FQ*_max_ expressed as stress), and (**F**) differences between muscles of the Nile crocodile. The error bars indicate the SD; results with asterisks show statistical differences between the muscles in our LME models.

Our results show some differences in performance between the different muscles in power output and contraction velocity. Table 2 summarizes the values and the means for muscle fiber bundles we measured from our Nile crocodiles. Bar graphs of the isometric stress, maximum power (in W L^−1^), maximum velocity of shortening (*V*_max_), normalized maximum power, optimal velocity of shortening at maximum power, and stress at maximum power compared between muscles are presented in Fig. 5. Several LME models were compared (see Materials and Methods section) to ensure that variance in the data was characterized using the simplest possible model. The resulting LME models that best characterize the variance with the lowest AIC (e.g., best goodness of fit, with penalizing the number of parameters) are presented in Supplementary data, Table S1. See the Discussion section for more explanation.

**Table 2:** Summation of the measurements and results for muscle physiology and mechanical performance in Nile crocodiles

	Body mass	Muscle length	Muscle mass	CSA	*σ*	*F_Q_*_max_	Max power	*V*_max_	*Q*_max_	V_@Qmax_	Stress at *Q*_max_	
Muscle	(kg)	(mm)	(mg)	(mm^2^)	(kPa)		(W L^−1^)	(s^−1^)	(s^−1^)	(s^−1^)	(kPa)	Fatigue
Rhm												
Average	3.58	32.6	45.8	1.62	218.0	0.28	66.3	4.27	0.320	1.17	61.4	78
SD	1.36	17.0	12.6	0.42	82.8	0.06	28.0	1.67	0.102	0.42	25.1	12
BB												
Average	3.98	29.4	64.6	2.12	183.0	0.34	73.6	3.88	0.420	1.27	61.2	85
SD	2.01	6.1	13.7	0.54	45.0	0.03	26.7	1.89	0.194	0.59	14.8	12
FTI4												
Average	3.37	45.3	62.2	1.29	227.9	0.32	145.5	6.44	0.626	1.98	72.1	56
SD	1.39	7.0	28.6	0.52	50.8	0.04	53.3	1.87	0.082	0.39	12.0	22

Averages and SDs presented. Shown: individual body masses, the length of the muscle, muscle mass, CSA, maximal isometric stress (*σ*), force normalized by *F*_IM_ at the maximum normalized power (*F_Q_*_max_), volume-normalized maximum power, normalized *V*_max_, normalized maximum power (*Q*_max_), velocity at maximum power (V@*Q*_max_), stress at maximum normalized power (stress at *Q*_max_), and rate of fatigue (“Fatigue” or fatigability; number of activations for a muscle to reach 50% of its initial isometric force).

### Muscle performance

We found wide variation in the isometric stress generated by the muscles. Both the Rhm and the FTI4 had similar average isometric stress measurements (218.0  and 227.9 kPa, respectively), but the measurements within the Rhm muscles ranged from as low as 108 kPa, up to 328.0 kPa (Supplementary data, Table S2). Variation across the three muscles led us to find no statistical differences in stress generation between muscle IDs (Fig. 5A, Supplementary data, Table S1: Isometric stress; MuscleID; *F*_stat_ < 1).

**Fig. 6:**
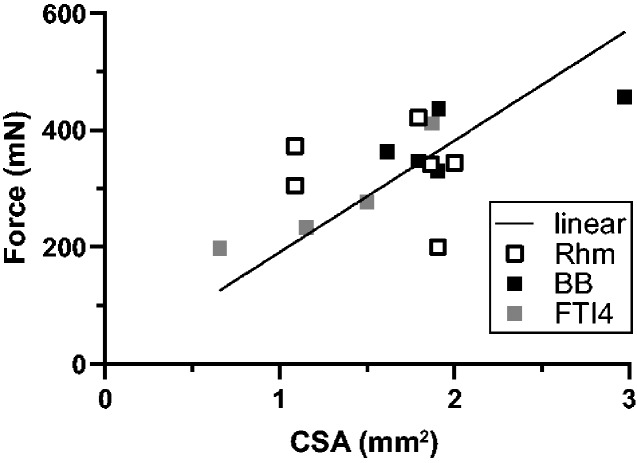
Maximal isometric force capacity (*F*_IM_) and muscle fiber cross-sectional area (CSA) relationship in Nile crocodiles. Force (mN) as a function of muscle fiber bundle CSA (mm^2^). The trendline “linear” is set to intercept the axes at 0, enforcing a proportional relation between CSA and F_IM_ at (CSA [mm^2^] · 191 [kPa] = F_IM_ [mN]; 95% confidence intervals = 164–217 kPa; *N* = 15; *P* < 0.0001).

The variation in isometric stress generation also showed in the poor relationship between force and CSA. Figure 6 summarizes the *F*_IM_s (mN) and CSA’s (mm^2^) across all preparations tested; most of the values lie within the narrow CSA range of 1–2 mm^2^. No more than a general trend of increased force with increased CSA was found. Figure 6 also shows the range of force variation for each muscle. The Rhm, in particular, showed a relatively wide range of force generation between muscle samples; all of the Rhm results appear within the 1–2 mm^2^ CSA zone, but they cluster poorly around the force–CSA relationship that the BB and FTI4 bundles seem to form.

The maximum power output (W L^−1^) did show a clear difference between muscles (Table 2). We find that the Rhm averaged around 66.3 W L^−1^, with some variation in two muscle samples of around 100 W L^−1^ and the rest closer to 50 W L^−1^. The BB had slightly less variation, but similar maximum power output average across the measurements (73.6 W L^−1^). Our statistical analysis showed that the primary variance was attributed to the muscles (Supplementary data, Table S1; Power [W L^−1^]; MuscleID; *F*_stat_ = 5.3), and secondarily by body mass (*F*_stat_ = 3.7). There was no statistically relevant difference between the Rhm and BB (Fig. 5B and Supplementary data, Table S1). The FTI4 stood out from the other two muscles, measuring double the average maximum power output vs. any of the others (145.5 W L^−1^). The maximum power output for the FTI4 tested higher than the Rhm in our statistical analysis, but not relative to the BB (Fig. 5B and Supplementary data, Table S1).

Peak normalized power (*Q*_max_) is independent of differences in muscle isometric stress and muscle size within a group of preparations and so is a measure of the intrinsic muscle power that allows for comparison of power output between the three muscles. The average normalized powers varied in a pattern similar to that depicted for power in W L^−1^, but there were clearly significant differences between the FTI4 and both Rhm and BB. The *Q*_max_ of the FTI4 was close to double the average value of the Rhm (Fig. 5D and Supplementary data, Table S1).

Measurement of the stress that generates maximum power can be calculated from the values of relative force at peak power (*F_Q_*_max_) and the maximum isometric stress. The stress at peak power (Table 2 and Fig. 5F) was slightly higher in the FTI4 than in the BB and Rhm, but was not statistically different relative to either.

The average values for *V*_max_ optimal velocity of muscle shortening at maximum power (V@*Q*_max_) showed similar general muscle ID dependency to the that shown among the average *Q*_max_ values, with those for the FTI4 being higher than the BB and Rhm muscles, and the BB and Rhm average *V*_max_ and V@*Q*_max_ being quite similar to each other (Table 2, Fig. 5C and E). The FTI4 average *V*_max_ and average V@*Q*_max_ were significantly higher than the values for BB and Rhm in our LME analyses. Differences in muscle bundle shortening speed, and not stress at peak power, appear to explain the greater average power in the FTI4 compared to the BB and Rhm muscles.

The rate of fatigue within Nile crocodile muscles showed a higher fatigability for the FTI4. Both the Rhm and BB were more resistant to fatigue, reaching 50% of their initial isometric force after 78 and 85 consecutive activations, respectively. The FTI4 was more easily fatigued; on average, half of the isometric force was reached after 56 activations. As part of the LME model analysis, we tested whether the rate of fatigue had an effect on the performance of the muscle bundles. No statistical influence of fatigue was found on any of our performance measures.

### Muscle immunostaining

Immunostaining was done to provide a qualitative picture of MHC-I and -II distribution in muscle bundles dissected directly adjacent to the bundles used for mechanics testing. We did not count fiber types partly because reliable identification of MHC-II subtypes and hybrids was not possible, and partly because the bundles that were stained varied in size (cross-section) and total fiber number. Also, the triton-X 100 skinned bundles will have been swollen and lacking both intracellular compartmentation and consistent collagen demarcation of fiber margins. Nevertheless, the actin–myosin lattice remains intact in relaxed skinned fibers and indeed remains functional in the presence of calcium and adenosine–triphosphate. The myosin heads are available for antibody binding in thin sections. We assumed that the MHC content of these small skinned muscle strips is representative of the larger intact bundles used for mechanics testing.

**Fig. 7:**
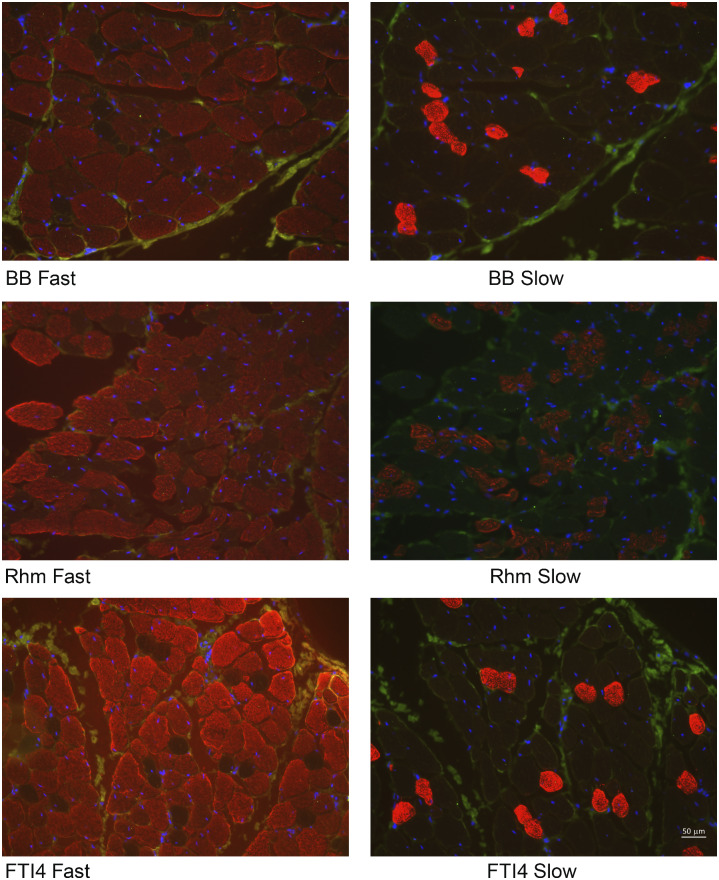
Representative images of immunohistochemical staining of Nile crocodile muscle fiber bundles (from “Croc 5”). Muscle ID (BB/Rhm/FTI4) is given followed by “Fast”/“Slow” for Types I or II staining. Blue highlights the Type I (slow-twitch) and red highlights the Type II (fast-twitch) fibers in the images, which all were viewed with ×20 magnification. For all other samples see Supplementary Material S1. Note 50-micron scale bar in bottom right panel.

A total of eight muscle fragments (three each of FTI4 and Rhm and two of BB) from three different crocodiles were cut and stained successfully. An example set of images (Fig. 7), where the muscles stained all came from the same crocodile, shows that bundles from all three muscle groups contained both MHC-I (slow-twitch) and MHC-II (fast-twitch) fibers. The FTI4 and BB cross-sections showed similar densities of MHC-I fibers, while the Rhm fragment tended to show greater MHC-I content. The BB and FTI4 comparisons were visually consistent across the subset of muscles sampled for immunostaining (see Supplementary Material S1 for images not shown in Fig. 7). The two Rhm samples that were sectioned had quite different densities of MHC-I (compare Fig. 7, Panels C and D), perhaps consistent with the variable degree of surface “pinkness” that we noted in particular the Rhm coloration during dissection.

## Discussion

The main aim of this study was to characterize and compare the mechanical performance of different appendicular muscle fiber bundles in Nile crocodiles (*C. niloticus*). Maximum isometric stress, power output, and contraction velocity were chosen because they are aspects of contractile performance particularly relevant to muscle function *in vivo* and whole-animal performance. F–L properties also help to establish the *in vivo* operating lengths of the various muscle groups (non-normalized data in Supplementary data, Table S2). Work-loop analyses informed by our study and data on crocodylian locomotor biomechanics could constitute a particularly informative follow-up study. Characterization of these properties allows comparison amongst muscles and across species, and informs the use of Hill-type muscle models in musculoskeletal simulations using crocodylians, sole survivors of the pseudosuchian lineage of the great Mesozoic radiation of Archosauria. The data we provide are most directly useful for studies of maximal performance, as our experiments involved maximal muscle stimulation that incurred maximal isometric stresses, contraction velocities, and powers; as well as fatigue estimates. However, Hill-type models can scale down to submaximal performance with some success (e.g., Zajac 1989; Millard et al. 2013), and emerging computational tools enable modeled muscles to incorporate some other influences such as activation levels (e.g., Cox et al. 2019).

**Fig. 8:**
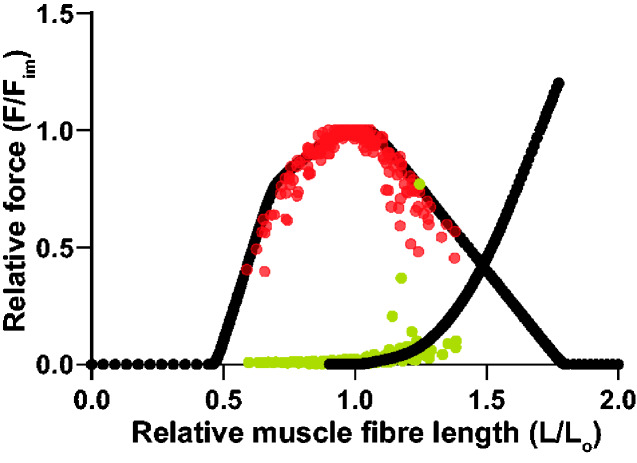
F–L data from Fig. 2 plotted against the Hill-type model curve from Millard et al. (2013). The X-axis scale from Fig. 2 has been modified by +1 L_0_ to match the model plot’s axis format. This qualitatively encouraging fit is analogous to that of other experimental data plotted in Fig. 3C of Millard et al. (2013) and has similar overall variability to those data.

Our data on isolated Nile crocodile muscle bundles revealed broad patterns, such as fitting a Hill-type model of F-L well. Specifically, Fig. 8 shows our F–L data from Fig. 2 plotted against the Hill-type model curve from Millard et al. (2013), popularly implemented in current versions of OpenSim musculoskeletal simulation software, revealing a qualitatively encouraging fit; analogous to (and overlapping with variability for) other experimental data plotted in Fig. 3C of Millard et al. (2013). There were also some relevant statistical differences among the performance measures. We found that the power-generating hindlimb FTI4 muscles had relatively high content (qualitatively) of fast (MHC-II) muscle fibers and average performance measures, such as estimated *V*_max_, the velocity at peak power, *Q*_max_, and peak power (in W L^−1^), that were 50–220% higher than those for the Rhm, which is a muscle presumably used more for stabilization of the appendicular skeleton than for powerful locomotion. The forelimb BB displayed characteristics that were broadly intermediate to the FTI4 and Rhm, with MHC-I and MHC-II contents (qualitatively) resembling those in FTI4, but with power measures only slightly greater than the Rhm. Overall, the muscle preparations demonstrated stress, power, and shortening velocity values on par with some high-performance muscles in terrestrial mammals (e.g., Close 1972; Seow and Ford 1991; Josephson 1993; Bottinelli et al. 1996; Medler 2002; West et al. 2013; Curtin et al. 2015; Wilson et al. 2018). Although amphibious, Nile crocodiles are nevertheless expected to exploit a capacity for high muscle performance, to support surprising bursts of speed on land including the use of asymmetrical gaits (Cott 1961; Hutchinson et al. 2019).

All three muscles studied were found to perform slightly differently with regards to the amount of power, stress, and velocity during contraction. Each of the three muscles sampled has different functions and anatomical locations within crocodiles. The Rhm seems to be involved in maintaining the position of (i.e., stabilizing) the Sc and supporting forelimb-based locomotion. Although it has been little-studied, the Rhm is thought to play a central role in stability and balance of the body (via its scapular insertion) during locomotion (Fujiwara et al. 2009; Baier and Gatesy 2013; Fujiwara 2018). The Rhm was the most difficult to dissect for the measurement apparatus and we noticed variability in the degree of “pinkness” in surface color across the Rhm muscle samples. Some of the variability in mechanical properties of our Rhm perhaps reflects variability in fiber type distribution (see below). The variation in force-CSA properties observed for the Rhm (Fig. 6) could be exaggerated by the architecture of the Rhm muscle and by its possibly more heterogeneous fiber-type arrangement. Dissecting a sample from a tapered, wide muscle that originates on the vertebrae and inserts broadly onto the Sc, and possibly expresses variable MHC isoform content along with its breadth, may have added to the variation, particularly considering the moderate size range of our Nile crocodiles. The BB is a forelimb protractor, likely primarily active during the swing phase to aid in limb recovery between stance phases (e.g., Meers 2003; Baier and Gatesy 2013). The FTI4 is a hindlimb retractor, presumably active during stance phase to play primarily antigravity and propulsive roles (e.g., Gatesy 1997; Allen et al. 2014; Cuff et al. 2019). Although the number of muscle bundles measured for each group was limited, the results are indicative of performance differences between these samples of Rhm, BB, and FTI4 that broadly align with their different whole-muscle roles during locomotion.

The F–V properties summarized in Table 2 and detailed in Supplementary data, Table S2 show considerable variability. Even the FTI4, which we can consistently classify as high power and speed compared with Rhm and BB, had a 2-fold range of power (in W L^−1^). However, coefficients of variation (CV = SD/mean) for the various performance measures across the three anatomical muscles were usually <0.4 and approached 0.5 for only the BB values of *Q*_max_ and *V*_max_. These CV values are in line with those seen in a previous comparison of skinned-fiber and intact-bundle performances of wild rabbit muscles, using the same tools and methods for collecting intact bundle mechanics, and using the same analysis approach (see Curtin et al. 2015). The combined CV for isometric stress, for example, across the 3 crocodile muscles (15 samples) was 0.30, compared to 0.42 for isometric stress across 2 rabbit hindlimb muscles (16 samples). These are still higher than CVs for numerous other sauropsid appendicular muscles (e.g., ∼0.10 in Nelson et al. 2004; ∼0.07 in Kohlsdorf et al. 2004).

Part of the variability in peak isometric stresses, and peak power in W L^−1^, is related to the errors in determining bundle CSA, fiber-length, and volume, owing to the presence of damaged and non-excitable fibers in small bundles dissected away from large whole muscles (discussed extensively in Curtin et al. 2015). While much of this tissue will have been removed from the surfaces of ethanol-fixed bundles before they were rehydrated and weighed, there would still have remained a variable component that contributed to bundle mass and volume. When comparing muscle powers, the mean *Q*_max_ is appropriate since the value is not dependent on (i.e., does not require) a measurement of bundle CSA or volume. If bundle size is a key factor contributing to performance variability, then the CV for power in normalized units (s^−1^) should be smaller than for power in W L^−1^; the FTI4 muscles clearly showed this effect, where the CV for *Q*_max_ values was 0.13 and 0.37 for power in W L^−1^. The CV values for Rhm and BB power were not as greatly affected, suggesting that factors in addition to bundle size, including possibly MHC isoform distribution, contributed more to variability in power amongst the Rhm and BB bundles than in the consistently fast-contracting and powerful FTI4 bundles.

While the sample size in this study likely contributed to within-group CV, numerous other factors are likely to also account for within- and between-group variability. In a study of rabbit muscles, animal body mass had no influence on muscle bundle F–V properties, but samples from male rabbits produced significantly greater peak isometric stress than females (Curtin et al. 2015). In this study, we also accounted for the potential influence of various known factors (e.g., muscle bundle length, animal body mass, and muscle bundle fatigability) on the variation within our results; we included them as fixed linear covariates within our LME models (Supplementary data, Table S1). Specifically, an effect of muscle length combined with body mass would potentially illustrate the influence of more mature, larger crocodiles relative to smaller juvenile individuals or vice versa. This led us to explore and quantify the effect of the body mass of our individual Nile crocodiles and effect of muscle length on the performance measures. Including both muscle length and body mass as potential covariates helped to distinguish between variation effects due to body size rather than actual differences in muscle performance or morphology. The outcomes, detailed in Supplementary data, Table S1, are summarized in Fig. 5 and confirm that the hindlimb FTI4 muscle was significantly faster and more powerful than both the forelimb BB and scapular Rhm muscles.

The maximum power generation in the hindlimb retractor muscles (FTI4) was twice that of the forelimb flexor (BB) and forelimb support (Rhm) muscles in Nile crocodiles (Table 2). The primary factor affecting the maximum power generation in Nile crocodiles found in our statistical analysis was the muscle ID (*F*_stat_ = 5.3); the effect of whether the muscle was the Rhm, BB, or FTI4. This was reflective of the higher power output of the FTI4 (145.5 W L^−1^ average), relative to the BB (73.6 W L^−1^) and Rhm (66.3 W L^−1^). Body mass had less effect (*F*_stat_ = 3.7), and no other factors clearly influenced our maximum power measurements in Nile crocodile muscles. The normalized maximum power measure (*Q*_max_) showed the same general pattern. The variation in *Q*_max_ was similar to peak power as well, although the SD around the mean *Q*_max_ for FTI4 was clearly reduced compared to that for the mean power in W L^−1^ and the mean *Q*_max_ for FTI4 was significantly different from both the BB and Rhm. Muscle ID was the primary source of variation (*F*_stat_ = 23.29) followed by body mass (*F*_stat_ = 12.11); muscle length had little effect (*F*_stat_ = 3.89). While peak stresses, and the stresses that generated peak power, were not different across the three anatomical muscles (Fig. 5A,F), the mean *V*_max_ and contraction velocity that generated peak power were significantly faster in FTI4 muscle (Fig. 5E,F). Our LME models showed that variation in contraction velocity was due to body mass (*F*_stat_ = 8.82) and muscle ID (*F*_stat_ = 7.02) but no other factors (Supplementary data, Table S1). The summary of performance measures (Fig. 5) indicates that it was chiefly faster muscle shortening velocity that accounted for the greater average power in the FTI4 muscles. The greater velocity and power in the FTI4 would seem to be intrinsic properties of the muscle compared with the Rhm and BB, possibly related entirely to the muscle fiber composition.

The maximum power generation in our Nile crocodile muscles was generally comparable to values in other tetrapods. For example, the maximum power of the M. iliofibularis in the desert iguana (*Dipsosaurus dorsalis*) ranged 42–154 W kg^−1^ over the temperature range of 22–42°C (Swoap et al. 1993); itself comparable to (albeit sometimes lower than) other lizard locomotor muscles (John-Alder and Bennett 1987; Curtin et al. 2005; James et al. 2015; Anderson and Roberts 2020). The M. iliotibalis from *Xenopus tropicalis* generated 60–100 W kg^−1^ of power in the temperature range of 24–32°C (James et al. 2012). Locomotor muscles of birds (including *in vivo*) can produce greater maximal powers >300 W kg^−1^ (Askew et al. 2001; Nelson et al. 2004; Ellerby and Askew 2007; Morris and Askew 2010) but other avian muscles act closer to the range we measured (e.g., 119 W kg^−1^, Dial and Biewener 1993; ∼60 W kg^−1^, Gabaldón et al. 2004,^ 2008^). Intact M. peroneus longus and M. extensor digiti V bundles from wild rabbits were able to produce average maximum power of 121.3 W L^−1^ (Curtin et al. 2015). These mammalian muscles were measured at 25°C, likely below their physiological optimum for power generation. In Nile crocodiles, muscle function for locomotion at 25°C would be commonplace, although it is likely, as in other ectotherm vertebrates, that muscle velocities of shortening and peak powers are temperature sensitive ∼25°C (Putnam and Bennett 1982; John-Alder and Bennett 1987; Swoap et al. 1993; Herrel et al. 2007). Muscles from various ectotherms increase peak power between 1.5- and 2-fold for a 10°C temperature-rise (Josephson 1993). A key response to higher temperature could be to increase capacity for sustained high-power contraction cycles, of the type that would support a prolonged *in vivo* burst of locomotion (James et al. 2012). Nile crocodiles possibly exploit such muscle-level enhancement of power generation during their regular movements between land and aquatic habitats, when body temperature will cycle with environmental temperature. Muscles, however, may operate closer to maximal force production than maximal power production (i.e., at low values of *V/V*_max_) especially if pennate and in series with tendon as in many distal muscles (Gabaldón et al. 2008) but our three crocodylian muscles were more parallel-fibered and less tendinous so maximal power output capacity may be more relevant, especially during activities involving maximal muscle activation; similar to some high-power flight muscles noted above for birds.

Average maximum isometric stress and *V*_max_ are also comparable to values obtained using other tetrapods (Table 3). Isometric stress (kPa) generation in crocodiles was most affected by muscle length (*F*_stat_ = 6.1), but not by body mass (Supplementary data, Table S1). However, this effect was likely dominated by the higher stresses clustering in the longer FTI4 muscle bundles (averaging 227.9 kPa, 45.3 mm) and the somewhat lower stresses within the shorter BB muscle bundles (averaging 183 kPa, 29.1 mm). The average maximum stress generated in Nile crocodile muscles was within the range of stress measurements in other non-avian sauropsid species. At 183 kPa, the Rhm is close to the isometric performance of M. iliofibularis fibers in western fence lizards (*Sceloporus occidentalis*) at 188 kPa (Marsh and Bennett 1986) or M. iliofibularis in the red-eared slider terrapin (*Pseudemys scripta elegans*) at 183 kPa (Mutungi and Johnston 1987). Our BB and FTI4 data are more similar to the 214 kPa in the fast-twitch glycolytic region of M. iliofibularis in the desert iguana (*Dipsosaurus dorsalis*) (Marsh 1988) and values for M. gastrocnemius in other lizards (Putnam and Bennett 1982; James et al. (2015); Table 3); or chicken M. pectoralis (e.g., 165–174 kPa; Reiser et al. 1996). Based on the small sample size of muscles, we measured and limited comparative literature for crocodiles (stresses similar to FTI4 for M. caudofemoralis longus in Seebacher and James 2008; Table 3), it seems that stress generation in Crocodylia might be on the higher end of the performance range within reptiles studied to date, but is still within typical variation (∼150–300 kPa) for vertebrate skeletal muscles (e.g., Medler 2002). Crocodylian values compare well with avian hindlimb muscle stresses (Nelson et al. 2004) but are higher than those often measured for some flight muscles (Table 3), suggesting further muscular specialization between such locomotor modes but some conservatism within them.

**Table 3:** Comparison with muscle biomechanical data from extant Sauropsida (Aves and nonavian)

Group	Common name	Species	Muscle (type)	Mass (g)	V_max_ (s^-1^)	*σ* (kPa)	Temp (°C)	Reference
Birds	King quail	*Coturnix chinensis*	Pectoralis (F)	45.7	26.00	130.9	40	Askew and Marsh (2001)
	Japanese quail	*Coturnix coturnix*	Anterior latissimus dorsi (F)	100	2.6	111	25	Alway (1994, 1995)
	European starling	*Sturnus vulgaris*	Pectoralis (F)	71.5	22.3	122.7	40	Biewener et al. (1992)
	Zebra finch	*Taeniopygia guttata*	Pectoralis (F)	13.1	9.8	167	40	Ellerby and Askew (2007)
	Budgerigar	*Melopsittacus undulatus*	Pectoralis (F)	42	5.6	220	40	
	Cockatiel	*Nymphicus hollandicus*	Pectoralis (F)	91.8	21.2	215	40	Morris and Askew (2010)
	Domestic chicken	*Gallus gallus*	Pectoralis (F), white	1500	4.66	165	15	Reiser et al. (1996)
			Pectoralis (F), red		2.59	174		
			Latissimus dorsi (F)		0.45	126		
	Wild turkey	*Meleagris gallopavo*	Gastrocnemius pars lateralis (T)	4000	13	271	38	Nelson et al. (2004)
			Fibularis longus (T)		14.80	257		
Non-avian Sauropsida	Western fence lizard	*S. occidentalis*	Iliofibularis (T)	13.7	21.9	187	35	Marsh and Bennett (1986)
	Desert iguana	*D. dorsalis*	Iliofibularis (T) FG, FOG, slow	55.8	7.5, 4.4, and 1.5	279	25	Gleeson and Johnston (1987)
	Desert iguana	*D. dorsalis*	Iliofibularis (T)	20	20	214	40	Marsh (1988)
	Desert iguana	*D. dorsalis*	Iliofibularis (T)	49.1	n/a	178	41	Putnam and Bennett (1982)
			Gastrocnemius (T)			262		
	Laurent’s whiptail	*Cnemidophorus murinus*	Iliofibularis (T)	58.4	n/a	137	40	
			Gastrocnemius (T)			182		
	Western fence lizard	*S. occidentalis*	Iliofibularis (T)	11.4	n/a	85	35	
			Gastrocnemius (T)			237		
	Southern alligator lizard	*Gerrhonotus mullicarinatus*	Iliofibularis (T)	25.4	n/a	135	28	
			Gastrocnemius (T)			237		
	Bosc’s fringe-toed lizard	*Acanthodactylus boskianus*	Caudofemoralis longus (T)	4.3	∼10	n/a	39	Curtin et al. (2005)
	Egyptian agama	*Trapelus pallida/mutabilis*	Caudofemoralis longus (T)	16.72	n/a	454	35	Herrel et al. (2007)
	Ruthven’s anole	*Anolis bonairensis*	Ambiens pars ventralis (T)	6	11.3	132	33.4	Anderson and Roberts (2020)
	Green anole	*Anolis carolinensis*	Ambiens pars ventralis (T)	6	6.0	179	28.2	
	Crested anole	*Anolis cristatellus*	Ambiens pars ventralis (T)	10	10.8	226	29.1	
	Striped anole	*Anolis lineatus*	Ambiens pars ventralis (T)	10	11.8	131	32.2	
	Brown anole	*Anolis sagrei*	Ambiens pars ventralis (T)	3	7.7	142	32	
	Pale-rumped skink	*Ctenotus regius*	Iliofibularis fast (glycol, T)	6.1	19.1	251	35	John-Alder and Bennett (1987)
	Striped skink	*Ctenotus robustus*	Iliofibularis fast (glycol, T)	13.1	20.1	223	35	
	Copper-tailed skink	*Ctenotus taeniolatus*	Iliofibularis fast (glycol, T)	6.0	19.7	215	35	
	Spotted-back skink	*Ctenotus uber*	Iliofibularis fast (glycol, T)	6.2	19.8	207	35	
	Thick-tailed skink	*Eremiascincus fisciolatus*	Iliofibularis fast (glycol, T)	16.0	16.2	238	25	
	Alpine water skink	*Sphenomorphus kosciuskoi*	Iliofibularis fast (glycol, T)	10.3	16.0	187	30	
	Eastern water skink	*Sphenomorphus/Eulamprus quoyi*	Iliofibularis fast (glycol, T)	26.9	18.2	264	30	
	Southern water skink	*Sphenomorphus/Eulamprus tympanum*	Iliofibularis fast (glycol, T)	17.9	16.2	193	30	
	(Rock-outcrop specialist calango)	*Tropidurus itambere*	Iliofibularis	8.0	N/A	165.8	35	Kohlsdorf et al. (2004)
	(sand dune specialist calango)	*Tropidurus psamonastes*	Iliofibularis	15	N/A	156.2	35	
	(generalist calango)	*Tropidurus oreadicus*	Iliofibularis	12	N/A	144.6	35	
	(17 lacertid lizard species; mean ± SD)	Lacertidae spp.	Iliotibialis	6.3 ± 4.4	N/A	322 ± 108	34	James et al. (2015)
	Red-eared terrapin (slider)	*Trachemys (Pseudemys) scripta elegans*	Iliofibularis fast (glycol, T, S)	305	5.5	184	15	Mutungi and Johnston (1987)
			Iliofibularis fast (oxo, T, S)		3.0	120.4	–	
			Iliofibularis slow (T, S)		1.3	70.6	–	
	Saltwater crocodile	*Crocodylus porosus*	Caudofemoralis longus (T, S)	727	N/A	230	30	Seebacher and James (2008)

Muscle types: T, terrestrial; F, flight; S, swimming. Mass (in g) refers to the body mass of the animal measured. Adapted from Table 1 in Medler (2002), with added data as referenced. Temp, temperature of experimental measurements. See Table 2 and Discussion for comparisons. “fast” or “slow,” and “glycol,” or “oxo” correspond to fast (F) or slow, glycolytic (G), and/or oxidative (O) fibers, when known. “N/A”, not available.

Maximum contraction velocity (*V*_max_) in Nile crocodile muscles (3.88–6.44 1.3 s^−1^; Table 2) is also comparable to data from some other reptiles. The values for crocodile BB, Rhm, and FTI4 are generally on par with, or somewhat faster than, *Pseudomys scripta* muscle velocities (1.3 s^−1^ in slow, 5.5 s^−1^ in fast glycolytic muscle) and a few lizards (Gleeson and Johnston 1987; Anderson and Roberts 2020), but slower than the ∼10–20 s^−1^ values determined in many muscles from smaller lizards (Table 3; but note higher temperature measurements in many cases). Rapid contraction velocities in leg muscles small lizards are not surprising relative to the appendicular musculature of a larger semi-aquatic ambush predator and negative allometry of muscle contraction velocities (Medler 2002). The maximum contraction velocity measured in Nile crocodile muscles is also comparable to values in the literature for rabbit muscles, at 25°C. The velocities (*V*_max_/L_0_) of the BB and Rhm muscles are just below a wild rabbit contraction velocity of 4.99 s^−1^ (Curtin et al. 2015), whereas the FTI4, a likely high-power generator *in vivo*, is only somewhat faster under zero load (6.44 s^−1^). The wild rabbit muscles reported on by Curtin et al. (2015) are not the primary motors for powerful locomotion, but they are involved in running and hopping; their performance is similar to that of fast IID-type psoas muscle fibers from New Zealand white rabbits (West et al. 2013). At physiological temperature, *V*_max_ values of mammalian muscles are likely to be twice as fast as when measured at 25°C (Faulkner et al. 1990; Josephson 1993; Bottinelli et al. 1996). The temperature sensitivity of *V*_max_ in M. iliofibularis from *Dipsosaurus dorsalis* seems lower than that of mammals (Marsh and Bennet 1986; Gleeson and Johnston 1987), but the velocity that generates peak power seems to double for 10°C temperature rises between 15°C and 45°C (Swoap et al. 1993). Our *V*_max_ data for crocodiles are much slower (by ∼2–6 times) than data from wild birds, including two distal hindlimb muscles of turkeys with stance phase activity (Table 3; see also Cuff et al. 2019), so the phylogenetic bracket for that important parameter is broad and deserves more exploration, in particular related to muscle fiber types (e.g., Fig. 7).

**Fig. 9:**
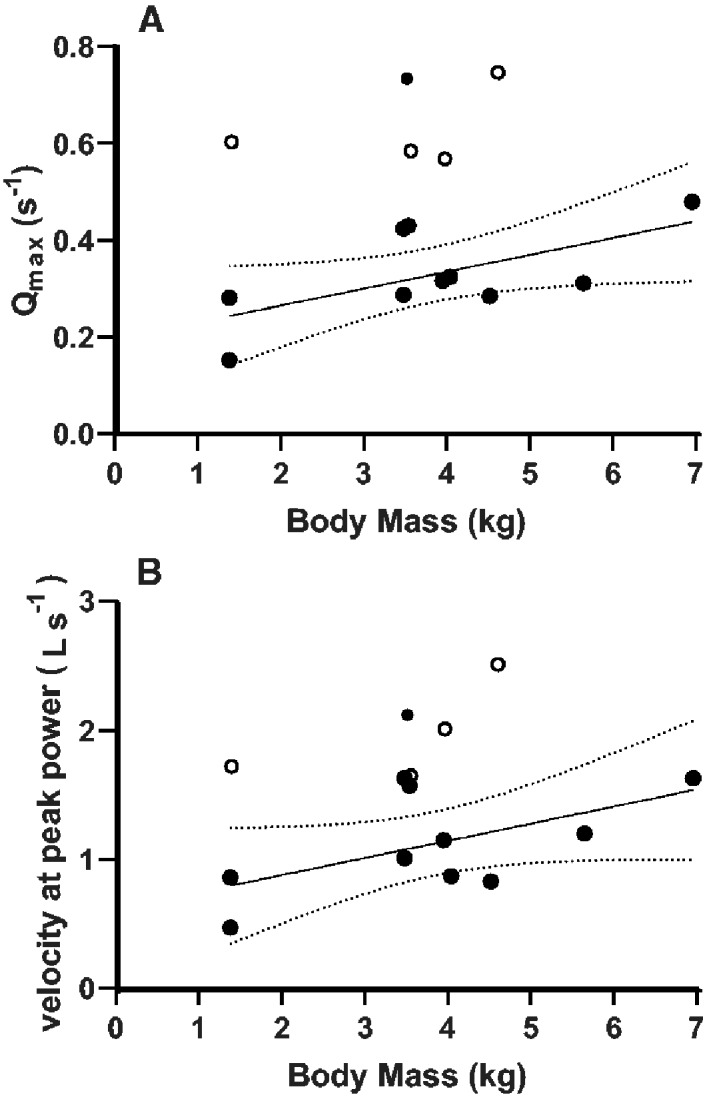
Ordinary least squares regressions of muscle contraction velocity-related parameters with body mass in Nile crocodiles, exploring sources of variation in the results. (**A**) There was a marginally insignificant (*P* = 0.0508) positive relationship between *Q*_max_ and animal body mass for most of the Rhm and BB muscles tested (filled symbols). One outlier Rhm muscle with a very high *Q*_max_ value was not included in the regression. The four *Q*_max_ results for FTI4 (open symbols) lay well outside the general relationship shown for the Rhm and BB. Dashed lines are the 95% confidence limits for the regression *Q*_max_ = 0.035 (body mass) + 0.195, in the body mass range of 1.38–6.96 kg. Including the outlier for Rhm produced the equation *Q*_max_ = 0.030 (body mass) + 0.253 (*P* = 0.343) but did not change the fundamental conclusions of this plot. (**B**) Velocity at peak power for Rhm and BB muscle bundles (solid symbols) of crocodile was marginally dependent on animal body mass. The regression V@*Q*_max_ = 0.13 (body mass) + 0.61 was for the same Rhm and BB body masses used in the regression in Fig. 9A. The slope was marginally insignificant (*P* = 0.08). Including the outlier for Rhm produced the equation V@*Q*_max_ = 0.12 (body mass) + 0.7595 (*P* = 0.22) but did not change the fundamental conclusions of this plot. The open symbols are for the four FTI4 muscle bundles tested.

The LME models showed that variation in velocity and power measures were almost universally significantly influenced by the factors body mass and muscle ID (Table 2 and supplementary data, Table S1). The muscle ID effect is clearly owing to the FTI4 being the most powerful and fastest-contracting of the muscles tested (Table 2 and Fig. 5). As FTI4 is presumably activated during stance phase for gravitational support and propulsion (e.g., Cuff et al. 2019), whereas Rhm and BB likely act in a more stabilizing role or during swing phase (Table 1), our results for muscle physiology broadly concur with expected locomotor roles. The marginally insignificant relationship between body mass and, for example, *Q*_max_ (Fig. 9A), showed that the FTI4 *Q*_max_ values plotted well above a more general relationship calculated for most of the Rhm and BB muscles tested. For Rhm and BB, linear regression predicted normalized muscle power to approximately double over the 7-fold range of body mass. There were not enough data to indicate whether the *Q*_max_ for FTI4 is similarly correlated with animal body mass. Normalized powers for both slow- and fast-twitch mammalian fibers correlated negatively with mammalian body mass over a very large interspecific 2.5 × 10^4^ range of body mass (Seow and Ford 1991). The increase in Rhm and BB normalized powers over only a 7-fold range of crocodile body masses was likely more related to animal maturation, and concurrently increased weight-bearing and increased locomotory performance (i.e., intraspecific scaling), than it was to a phenomenon that could be directly compared with interspecific size-scaling of muscle power. Velocities of muscle shortening for Rhm and BB muscles showed a similar marginally insignificant relationship to body mass (Fig. 9B), as did the peak rates of muscle force development (data not shown), suggesting that there was a greater density of fast-twitch Type II fibers in the muscle bundles dissected from the larger crocodiles. We did not sample the muscles comprehensively enough in our first-time analysis of muscle MHC-I and MHC-II isoforms to confirm a size-dependent shift in fiber-type distribution. There may be other concurrent changes with growth that complicate any such shift. Moreover, the patterns in Fig. 9 (which were relatively insensitive to inclusion/exclusion of the one Rhm outlier point) require more measurements over a larger range of masses, to further test the hypothesis that there are maturation-dependent changes in muscle fiber-type distribution. Such a finding would nevertheless be consistent with the tendency for greater Type II myosin expression in mammalian muscles during the early period of body maturation (Schiaffino and Reggiani 2011).

Immunohistochemical staining of the Nile crocodile muscles also concurred broadly with the observed differences in muscle bundle power, velocity, and average fatigability, in the sense that the density of small-diameter slow-twitch (MHC-I) fibers appeared relatively low in the FTI4 muscles and relatively high in the Rhm muscles. The relatively high MHC-I content of the Rhm is evident from representative images (Fig. 7), where we had successfully cut and stained small skinned fiber bundles of all three anatomical muscles from the same animal. While the BB and FTI4 muscles clearly had a similar number of MHC-I fibers, the overall number of fibers stained was somewhat higher in the FTI4 cross-section, suggesting a lower overall content of MHC-I. Quantifying MHC isoform distribution requires greater sampling and analysis of crocodile muscles. A key caveat is that we show cross-sections of small muscle fragments taken from muscle bundles which themselves were sub-sectioned from the overall FTI4, Rhm, and BB muscle masses. Nevertheless, this preliminary analysis does generally indicate that the distribution of fiber types seems to have matched the morphology and presumed function of each muscle within the Nile crocodile appendicular system.

Large-diameter fast-twitch (MHC-II) fibers should be most beneficial during rapid and powerful limb motion as in the FTI4, whereas small-diameter slow-twitch (MHC-I) fibers are likely more prevalent in muscles involved with stability and support of the limb, such as in the Rhm. The BB muscles clearly align with the physiological capacities of the Rhm (Figs. 5 and 8) but BB may have Types I and II fiber distributions more like that of the FTI4. The suspected swing-phase activity of the BB muscle during locomotion rather than overt stance phase power generation seems consistent with these mechanics and MHC contents. Additionally, the role for subtypes of MHC-II isoforms in BB is possibly a factor, since the MHC-IIA isoform is thought to convey an intermediate level of speed, power, energetics, and fatigability to muscles (Schiaffino and Reggiani 2011). Robust future measurement of MHC contents in these muscles would require sampling more of the muscle volume and development of the immunostain assays to include detection of the sub-type isoforms of MCH-II. Sampling from a wide range of animal sizes/ages would help address the idea that maturation influences the distribution of all the muscle MHC isoforms and of possible hybrid-isoforms.

## Conclusion

As extant members of the archosaur lineage, crocodylians can help provide a frame of reference for the evolution of muscle performance in early dinosaurs and their relatives. Using our data and data from the literature, we can now more exactly define a range of contraction velocities and power generation in appendicular muscles from extant members of both archosaurian lineages. These data support the inference that most archosaurian muscles measured to date have fairly consistent parameters (e.g., Tables 2 and 3); especially maximal isometric stress; except in regards to muscle contraction velocity and power, as expected for physiological differences such as fiber types. Through this exclusive data set on nine Nile crocodiles, combined with existing data on muscle activity patterns (Cuff et al. 2019), our understanding of the muscular performance impacting terrestrial locomotor ability of archosaurs is now more complete. This in turn will allow for more accurate comparisons of archosaur locomotor dynamics and more informed musculoskeletal modeling of biomechanical computer simulations in the future, and should inspire more investigation of the influence of muscle fiber types on velocity-related parameters of muscular biomechanics and physiology.

## Supplementary Material

obaa038_Supplementary_Data
