# Supplementary material for: Appendicular Muscle Physiology and Biomechanics in Crocodylus niloticus
Source: Integr Org Biol. 2020 Nov 5;2(1):obaa038. doi: 10.1093/iob/obaa038 (PMC7810574; doi:10.1093/iob/obaa038)
Supplement: obaa038_Supplementary_Data [file obaa038_supplementary_data.zip › Table_ST2_Supplemental.docx]

|  |  |  | **Body mass** | **Muscle length** | **Muscle mass** | **CSA** | **σ** | **Isometric force** | **Max Power** | **V_max_** | **Q_max_** | **V @ Qmax** | **σ at Q_max_** | **Fatigue** |
| --- | --- | --- | --- | --- | --- | --- | --- | --- | --- | --- | --- | --- | --- | --- |
|  |  |  | **kg** | **mm** | **mg** | **mm^2^** | **kPa** | **mN** | **W L^-1^** | **s^-1^** | **s^-1^** | **s^-1^** | **kPa** |  |
| Nile  crocodile  muscle | Rhm |  | 5.65 | 67.0 | 36.3 | 1.09 | 328.0 | 355.1 | 102.0 | 4.80 | 0.311 | 1.20 | 84.8 | 57 |
|  |  |  | 3.48 | 25.0 | 50.5 | 1.91 | 108.6 | 206.2 | 46.0 | 6.22 | 0.424 | 1.63 | 28.3 | 85 |
|  |  |  | 3.48 | 28.0 | 59.4 | 2.00 | 164.8 | 323.0 | 47.3 | 3.46 | 0.287 | 1.01 | 46.9 | 71 |
|  |  |  | 1.38 | 23.5 | 27.1 | 1.09 | 291.3 | 311.9 | 44.2 | 1.49 | 0.152 | 0.47 | 94.8 | 83 |
|  |  |  | 3.95 | 29.1 | 57.5 | 1.86 | 177.6 | 326.4 | 56.1 | 4.18 | 0.316 | 1.15 | 48.6 | 88 |
|  |  |  | 3.54 | 23.0 | 43.7 | 1.79 | 237.6 | 421.8 | 102.1 | 5.46 | 0.430 | 1.57 | 65.1 | 81 |
|  |  | Average | 3.58 | 32.6 | 45.8 | 1.62 | 218.0 | 324.1 | 66.3 | 4.27 | 0.320 | 1.17 | 61.4 | 78 |
|  |  | SD | 1.36 | 17.0 | 12.6 | 0.42 | 82.8 | 70.1 | 28.0 | 1.67 | 0.102 | 0.42 | 25.1 | 12 |
|  | BB |  | 6.96 | 39.5 | 80.0 | 1.91 | 227.6 | 432.2 | 108.9 | 5.78 | 0.479 | 1.63 | 66.8 | 89 |
|  |  |  | 3.48 | 29.0 | 70.2 | 2.28 | 124.8 | 280.9 | 91.7 | 6.11 | 0.735 | 2.14 | 42.8 | N/A |
|  |  |  | 1.38 | 23.0 | 72.5 | 2.97 | 147.7 | 434.0 | 41.5 | 2.77 | 0.281 | 0.86 | 48.0 | 89 |
|  |  |  | 4.04 | 27.0 | 51.3 | 1.79 | 196.1 | 345.0 | 63.6 | 2.34 | 0.324 | 0.87 | 73.4 | 68 |
|  |  |  | 4.52 | 28.6 | 49.0 | 1.62 | 218.8 | 349.8 | 62.1 | 2.42 | 0.284 | 0.83 | 74.8 | 95 |
|  |  | Average | 3.98 | 29.4 | 64.6 | 2.12 | 183.0 | 368.4 | 73.6 | 3.88 | 0.420 | 1.27 | 61.2 | 85 |
|  |  | SD | 2.01 | 6.1 | 13.7 | 0.54 | 45.0 | 65.0 | 26.7 | 1.89 | 0.194 | 0.59 | 14.8 | 12 |
|  | FTI4 |  | 4.59 | 51.5 | 36.2 | 0.66 | 300.6 | 195.8 | 224.6 | 8.78 | 0.747 | 2.52 | 89.2 | 34 |
|  |  |  | 1.38 | 37.0 | 45.2 | 1.15 | 204.8 | 232.8 | 123.5 | 5.00 | 0.603 | 1.73 | 71.5 | 69 |
|  |  |  | 3.95 | 50.7 | 100.5 | 1.87 | 221.8 | 410.8 | 126.3 | 7.10 | 0.569 | 2.02 | 62.5 | 81 |
|  |  |  | 3.54 | 42.0 | 66.8 | 1.50 | 184.4 | 271.6 | 107.8 | 4.86 | 0.585 | 1.66 | 65.0 | 41 |
|  |  | Average | 3.37 | 45.3 | 62.2 | 1.29 | 227.9 | 277.8 | 145.5 | 6.44 | 0.626 | 1.98 | 72.1 | 56 |
|  |  | SD | 1.39 | 7.0 | 28.6 | 0.52 | 50.8 | 94.0 | 53.3 | 1.87 | 0.082 | 0.39 | 12.0 | 22 |
